# Mesenchymal Stem Cell Senescence during Aging:From Mechanisms to Rejuvenation Strategies

**DOI:** 10.14336/AD.2023.0208

**Published:** 2023-10-01

**Authors:** Xinchen Jiang, Wenshui Li, Lite Ge, Ming Lu

**Affiliations:** ^1^The National & Local Joint Engineering Laboratory of Animal Peptide Drug Development, College of Life Sciences, Hunan Normal University, Changsha, China.; ^2^Hunan provincical key laboratory of Neurorestoratology, the Second Affiliated Hospital, Hunan Normal University, Changsha, China.; ^3^Department of Neurology, Second Xiangya Hospital, Central South University, Changsha, 410011, China, Changsha

**Keywords:** mesenchymal stem cells, aging, cell transplantation therapy, cell senescence, rejuvenation strategy, autologous MSCs transplantation

## Abstract

In cell transplantation therapy, mesenchymal stem cells(MSCs)are ideal seed cells due to their easy acquisition and cultivation, strong regenerative capacity, multi-directional differentiation abilities, and immunomodulatory effects. Autologous MSCs are better applicable compared with allogeneic MSCs in clinical practice. The elderly are the main population for cell transplantation therapy, but as donor aging, MSCs in the tissue show aging-related changes. When the number of generations of in vitro expansion is increased, MSCs will also exhibit replicative senescence. The quantity and quality of MSCs decline during aging, which limits the efficacy of autologous MSCs transplantation therapy. In this review, we examine the changes in MSC senescence as a result of aging, discuss the progress of research on mechanisms and signalling pathways of MSC senescence, and discuss possible rejuvenation strategies of aged MSCs to combat senescence and enhance the health and therapeutic potential of MSCs.

## 1.Introduction

Since age-related diseases are a widespread problem with profound socioeconomic and health consequences, a better understanding of aging would allow us to guide public policies to minimize its adverse impact on the healthcare system and society. Mesenchymal stem /stromal cells (MSCs) are a heterogeneous group of stromal cells derived from the mesoderm or ectoderm, which possess potent multipotency and regenerative characteristics. Emerging scientific evidence highlights the importance of MSCs in maintaining tissue homeostasis, preventing aging-related diseases, and providing clinically relevant cellular therapies. Recent studies have examined the phenomenon of aging and its underlying factors. Human aging is primarily caused by a reduced ability of adult stem cells to reproduce and regenerate. Therefore, the senescence of MSCs represents a significant barrier to the development of cellular therapies.

Friedenstein first described MSCs from bone marrow in 1970 [1]. The presence of alternative sources has recently been identified, such as adipose tissue [2], umbilical cord blood[3], dental pulp[4], endometrium [5], peripheral blood[6], periodontal ligament [7], placenta [8], synovial fluid [9], and the olfactory mucosa [10]. Evidence has shown that MSCs can exist throughout the entire body's vascularized tissues [11]. MSCs exhibit high proliferative ability, multipotency, also possess immunomodulatory properties, and paracrine function [12]. In addition to these factors, MSCs are also free of ethical concerns associated with embryonic stem cells, making them a perfect candidate for cell therapy, regenerative medicine, and tissue engineering applications. In recent years, MSCs have increasingly been used for the treatment of traumatic and degenerative conditions [13].

As soon as the number of cell divisions reaches Hayflick's limit, Cellular senescence promotes the aging and dysfunction of organisms [14]. It is the function of MSCs to replenish dying cells in order to maintain the normal operation of tissues, as well as to regenerate damaged tissues in several types of tissues [15]. Accordingly, MSCs play an important role in delaying the aging process of organs and tissues. There are many senescence-specific phenotypes associated with the expansion and culture of MSCs, including morphological changes, decreased proliferation activity, and unbalanced biological functions during the expansion and culture process [16]. In addition, the targeted patients of autologous MSCs transplantation therapy are most commonly senior citizens [17]. According to research, MSCs undergo aging-related changes throughout their lifespan, affecting their ability to survive transplantation [18]. It has been reported that many studies have focused on the function of MSCs in anti-aging and have rarely explored the senescence of MSCs themself. Numerous studies indicate that aged MSCs are not only affected by intrinsic factors, but also by the niche of aging tissues [19].The MSC niche is defined as a specialized microenvironment containing cells, cytokines, and the extracellular matrix that is responsible for maintaining the balance between self-renewal, quiescence, and lineage fate commitment in MSCs [20]. A tissue micro-environment surrounding MSCs is widely referred to as MSC niches due to these origin-dependent characteristics [21]. MSC niches undergo strong transcriptional changes as they senescent [22]. For example, the MSC niche produced by bone marrow stromal cells were compared with those produced by young versus elderly. In addition to decreasing MSC niche fibrillar organization and mechanical integrity, BM-MSC proliferation and responsiveness to growth factors decreased as donor age increased [23]. Senescent cells secrete SASP to create a toxic cellular microenvironment and promote additional cellular senescence through the activation of cytoplasmic signaling pathways [24]. Although cell senescence is primarily a defense mechanism, senescent cells accumulate with aging, promote tissue dysfunction through SASP, and trigger senescence in adjacent cells through paracrine mechanisms. It is possible to effectively maintain MSC property by simulating the MSC niche in culture.

**Figure 1.**
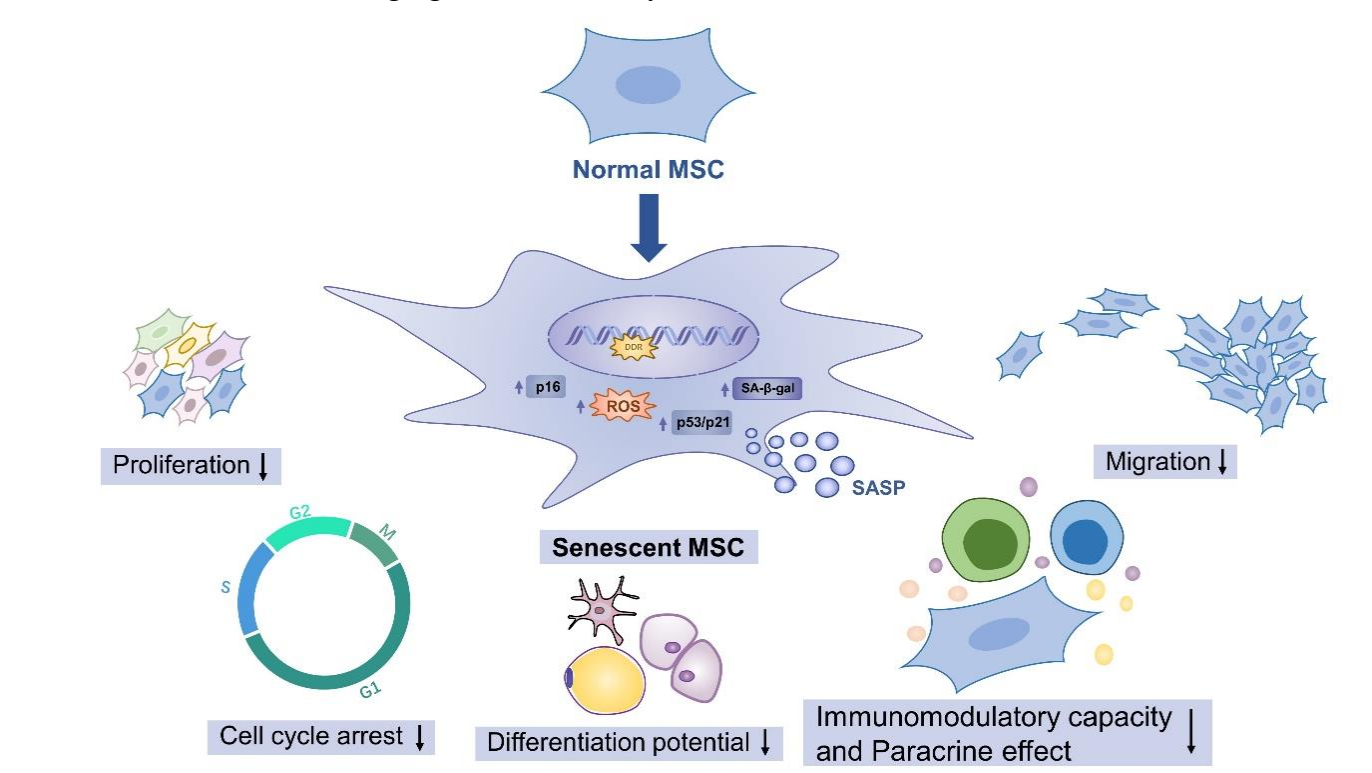
Age-dependent changes in MSCs during aging. During aging, MSCs become flat and broad with reduced proliferation and migration capacity, cell cycle arrest (p16 upregulation), oxidative stress injury (ROS increase), SA-β-gal and SASP increased. At the same time, the characteristics of stem cells are changed, and their differentiation will also be biased. MSCs also displayed reduced immunomodulatory capacity and paracrine effect.

In addition to their potential as therapeutic agents, MSCs have been discussed in terms of their role in regulating tissue homeostasis, tissue repair, and the development of diseases during aging. As of yet, no safe and effective stem cell therapy has been found that can slow or reverse the aging process of tissue. This review summarizes these changes in morphology, biology, and stem-cell properties observed in senescent MSCs. Then we focused on the mechanisms that lead to MSC senescence. Finally, we discussed how to reverse or rejuvenate MSC senescence to improve stem cell therapy and lifespan extension.

## 2.Defects in MSCs and MSC niches during aging

In aging, physiological functions are gradually disrupted, causing age-related diseases of impaired function, which ultimately result in death. MSC function declines during aging, resulting in deterioration of tissue repair and function [25]. Cells respond to aging (organism age or passage through a culture) which causes cells to slow down proliferation, diffuse, and acquire a secretory type to counter cumulative damage or changes [26]. Hayflick showed in 1961 that normal human fibroblasts have limited ability to divide cells before entering irreversible growth arrest known as replicative senescence [27]. MSCs, as well as other adult stem cells, are subject to the detrimental effects of aging. MSCs are also impaired during aging, thereby reducing their beneficial effects on cell transplantation [28]. Based on some research, the aging of donors and the increasing number of passages in vitro will cause some defects in MSCs. In general, defects in MSCs and MSC niches during aging can be seen in the following ways (Fig. 1).

### 2.1 Age-dependent changes in MSCs and MSC niches during aging

#### 2.1.1 Ability to produce committed progeny of MSCs reduced

Recently, cell-based approaches have been used to investigate MSCs decline during aging, when a variety of techniques are employed, including RNA sequencing (RNA-seq) and fluorescence-activated cell sorting (FACS). The imaging analysis indicates, for example, that MSCs began to enlarge at p5, resulting in a 4.8-fold increase when compared to p1, and that telomeres shortened at a constant rate during culture [29]. As far as human MSC markers (CD73, CD90, CD105) are concerned, the researchers were not able to identify any differences between young and older BM-MSCs. As the MSCs age, however, their characteristic spindle-like shape is lost, and they become flat and broad with reduced proliferation and migration capacity [30]. It was reported in 1999 that MSCs possess the capacity for tri-lineage differentiation, including osteogenesis, chondrogenesis, and adipogenesis [31]. The adipogenic and osteogenic potential of AD-MSCs, as well as skin from both adults and neonates, was found to differ significantly between these tissues [32]. MSCs exhibit morphological and functional changes as they age. According to Zhang and colleagues, MSCs have decreased proliferative and migratory potential as they age as well as differentiation capacity. Additionally, MSCs from elderly individuals were significantly more apt to express ATP and stage-specific embryonic antigen-4 (SSEA-4), as well as reactive oxygen species (ROS) intracellularly [33]. In vitro and in vivo, senescent MSCs are also significantly impaired in their ability to support the formation of vascular networks [34]. What’s more, Efimenko and colleagues analyzed AD-MSCs are less angiogenic potential in older patients due to a decrease in the secretion of proangiogenic factor [35]. Therefore, aged MSCs show related morphological and biological changes, including morphological enlargement and flattening, reduced proliferation and migration capabilities, and angiogenic potential affected. Based on these data, it appears that MSCs undergo an aberrant transition from progeny to migrat MSCs during aging. In 2003, Shi and colleagues published a study to identify MSC niches, suggesting that MSCs might reside first in the tissue microvasculature [36]. According to Geibler and colleagues, young MSCs always displayed greater migration potential than their older counterparts [37]. The evidence suggests that senescent MSCs do not respond to injury signals as well, and this characteristic may be related to aging-associated MSC niches.

#### 2.1.2 Age-dependent changes in paracrine factors and MSC niches

The paracrine property of MSCs plays an important role in cell therapy as well. Mancini and colleagues found that MSCs from elderly individuals displayed an inflammatory secretome containing increased levels of interleukin-6 (IL-6), IL-8/CXCL8, and monocyte chemoattractant protein-1(MCP-1/CCL2) [38]. Particularly, these senescent cells produced high levels of IL-6, one of the most prominent SASP factors [39]. Senescence-associated secretory phenotype (SASP) refers to the secretory phenotype that occurs in some senescent cells as a result of the increased secretion of pro-inflammatory cytokines, chemokines, proteins that damage tissues as well as factors that affect the function of stem and progenitor cells as well as growth factors. It has been demonstrated that pigment epithelium-derived factor (PEDF) expression is dramatically increased in BM-MSCs from aged mice compared to young mice, and that PEDF plays an important role in regulating MSCs' ability to protect against myocardial infarction (MI) [40]. As compared with hUC-MSCs at p2, p8 hUC-MSCs exhibited a reduced potential for proliferation, differentiation, immunoregulation, and secretion. Despite significantly suppressing the proinflammatory Th1 and Th17 populations at p2 and p5, hUC-MSCs at p8 were unable to do so. At p2 and p5, higher levels of growth factors, cell adhesions, and anti-inflammatory factors were observed than at p8 in terms of paracrine mechanisms [41]. According to Jin and colleagues, aged hUCB-MSCs secreted monocyte chemoattractant protein-1 (MCP-1) as a major component of the SASP and reinforced senescence through its cognate receptor chemokine receptor 2 via the ROS-p38-p53/p21 signaling cascade. The activated the p53 tumor suppressor (p53) increased the secretion of MCP-1 [42]. The microvesicles from MSCs (MSC-MVs) contain markers that are associated with MSCs, such as CD29, CD44, CD73, CD90, and CD105. Researchers have reported that old rat MSC-MVs contain unique miRNAs that inhibit the epithelial-mesenchymal transition mediated by TGF-1 [43]. RNA sequencing analysis revealed the same trend of decreasing miRNA levels with an increasing number of passages in both MSCs and MSC-MVs. Several genes highly expressed in senescent MSCs and their corresponding MSC-MVs are involved in the regulation of diseases associated with senescence [44]. Based on these findings, MSC-MVs are identified as a key contributor to the senescence-associated secretory phenotype of MSCs.

### 2.2 Accumulation of aging markers

During aging, transcriptomic and proteomic signatures associated also accumulate in MSCs. The accumulation of cellular damage and age-related markers may result in changes in transcriptional profiles. Researchers have identified senescence-associated-β-galactosidase (SA-β-gal) which has been found in aged tissues of various mammals as the first senescence biomarker. Cellular senescence was observed in aged BM-MSCs, as well as decreased proliferative capacity and paracrine function as compared to the BM-MSCs from young donors [28]. Another striking feature of senescent cells is their increased expression of cell cycle-inhibitory proteins (also known as cyclin-dependent kinase inhibitors). The most prominent cyclin-dependent kinase inhibitor involved in senescent cell accumulation is p16^INK4A^ (p16). Furthermore, p16 expression correlates strongly with aging, and measuring cellular p16 levels may be useful for both determining the biological aging of an organism and detecting cellular senescence. Senescence and apoptosis programs focus on key components, including activation of the p53 pathway, so resistance to apoptosis in senescent cells may also depend on its levels and activity. Furthermore, the expression of the tumor suppressor protein p53 and its pathway genes, p21 and Bcl-2-associated X protein (BAX), increased during aging [45]. Aging increases cellular senescence, which can be characterized by an increase in SA-gal-positive cells, an increase in mitochondrial-specific ROS production, and the expression of p21 [46, 47]. MSCs from the old exhibited significantly higher levels of ROS, and expression of ATP and SSEA-4 was significantly reduced[30]. Consequently, the levels of senescence biomarkers, such as SA-β-gal activity, and expression of pro-aging factors such as p16, p53, p21, and production of SASP and ROS gradually increased with MSC senescence.

### 2.3 Abnormal fate of MSCs with aging

As a special type of stem cell, MSCs possess the differentiation potential, immunomodulatory capacity, and paracrine effect. It is possible for subsets of MSCs to exhibit abnormal behavior as they age. Results mostly showed that MSCs may become senescent, excessively activated, or undergo an abnormal differentiation process with increased donors age and passage numbers [48]. Senescent MSCs, for instance, undergo irreversible arrest in their cell cycle and secrete cytokines and other proteins during aging [49]. MSCs are generally thought to be prone to self-renewal and reduced differentiation if these epigenetic regulators are partially lost. For example, the differentially regulated proteome characterized shows proteins of hBM-MSCs were differentially expressed with aging[50]. Similarly, the differentiation bias of some MSCs might be affected by aging. Aging causes a decrease in the commitment of BM-MSCs to the osteoblast lineage and an increase in the commitment to the adipocyte lineage [51]. BM-MSCs from aged mice also displayed a similar switch [52]. A study by D'Ippolito and colleagues has found that the number of hBM-MSCs with osteogenic potential decreases early in aging, which may explain why bone formation decreases with age [53]. An effort is being made to find new approaches to tap pristine reservoirs of MSCs in order to regenerate tissues during aging. It will be important to understand MSCs heterogeneity during aging. Comparing the global gene expression of young and aged hBM-MSCs, researchers observed decreased expression of cytokine and chemokine receptors in aged MSCs [18]. The role of microRNAs (miRNAs) in MSC aging was investigated using a genome-wide approach using MSCs from adipose tissue and bone marrow from both young and elderly donors. There was a significant increase in nuclear factor kappa B(NF-κB), myc, and interleukin-4 receptor mRNA levels in bone marrow and adipose tissues when cells were aged[54]. Therefore, property of MSCs declines with aging. What’s more, an examination of BM-MSCs from old and young rats revealed 868 differentially expressed genes (DEGs) that were up-regulated and 2006 that were down-regulated. A high expression of DEGs might be involved in cell differentiation and growth factor binding, whereas down-regulated DEGs appear to be involved in metabolism [55]. Circular RNAs(circRNAs) may compete with microRNAs (miRNAs) for the ability to stabilize or translate target RNAs, and thereby affect gene expression. Analyzing BM-MSCs from aged and young rats. These results indicate that 4,229 circRNAs play a role in MSC senescenc. There are 29 differentially expressed circRNAs in the aged group, of which four are upregulated and 25 are downregulated compared to the young group [56]. As a result of these studies, novel therapeutic targets for ameliorating age-related phenotypes were identified. Combined, aged MSCs are characterized by a reduction in number and function. The specific changes in MSCs with aging may vary depending on the source of MSCs. Thus, it is important to determine which senescence characteristics of MSCs are critical for tissue repair and disease treatment, as well as how to assess their senescence degree.

## 3. Molecular mechanisms of MSC senescence during aging

### 3.1 Epigenomic alteration

In MSCs, DNA methylation changes with aging, accompanied by transcriptional heterogeneity, suggesting epigenetic drift. Several epigenomic changes may be able to link MSCs to external stimuli. It is known that cellular DNA changes under complex chemical and physical environments. These changes are collectively referred to as DNA damage, while the response to maintain the integrity of the genome to counteract the adverse consequences of DNA damage is called DNA damage response (DDR). The DDR network senses and initiates mutation repair, hence preventing damage accumulation. When DNA damage persists, prolonged DDR activates the tumor suppressor p53 and the cell cycle kinases (CDK) inhibitor p21. The activation of p21 induces mitochondrial dysfunction and ROS production via p38^MAPK^. As a result, ROS increases DNA damage and maintains persistent DDR.

During aging, chromatin state and regulation undergo a number of changes. According to a comprehensive analysis of MSC senescence, chromatin accessibility increased, and transcription increased overall. For example, aged MSCs can be observed from the inhibition of DNA synthesis, p21, and p16 protein expression, and the elevation of SA-β-gal activity and often exhibit increased ROS, which once mutated in genes involved in DNA damage, can lead to premature aging [57]. Aging affects many aspects of chromatin state and regulation. For example, Jin and colleagues found that in aged hUCB-MSCs, MCP-1 played a dominant role in the SASP of aged hUCB-MSCs. C-C chemokine receptor type 2 (CCR2) activation by MCP-1 enhanced senescence in an autocrine and paracrine manner by activating ROS-p38-MAPK-p53/p21 signaling cascades. As BMI1 levels decreased during aging, the epigenetic status of MCP-1 changed, including the loss of H2AK119Ub, resulting in derepression of MCP-1 [42]. In order to maintain a stable senescent phenotype, it is important to consider the relationship between epigenetic regulation and SASP. Regulation of SASP by DDR may be one of the pathways by which DDR causes MSC senescence. The results discussed above indicate that MSC senescence is closely tied to DNA damage and related signaling pathways.

In MSCs, chromatin changes are affected by telomerase length and may play a role in mediating the effects of external properties and biophysical properties on MSCs as they are aging. As telomeres shorten with every cell division, cell cycle arrest and apoptosis may occur in MSCs. When telomeres reach a critical length, cellular senescence occurs[58]. For example, Samuelraj and colleagues analyzed the telomere lengths of hBM-MSCs from p1 until p15 at seven different passages and found that there was a decrease in the relative length as the MSCs underwent progressive cell divisions [59]. Furthermore, human fetal membrane-derived MSCs from mothers over the age of 40 had shorter telomeres and higher telomerase activity than those from mothers under the age of 40[60]. Similar changes have been made to human AD-MSCs and BM-MSCs from aged patients as well [35, 61]. As the catalytic core of telomerase, down-regulation of telomerase reverse transcriptase (TERT) inhibited cell proliferation and promotes senescence and apoptosis in MSCs, while TERT up-regulation promotes cell proliferation and reduces senescence and apoptosis [62]. Thus, targeted regulation of telomere length and telomerase activity is a promising research direction for the prevention of MSC senescence. Overall, these findings indicate the crucial role epigenomic changes play in long-term changes during aging as well as their ability to integrate extrinsic stimuli.

### 3.2 Loss of proteostasis

Autophagy is a tightly regulated non-specific degradation pathway that maintains cellular homeostasis by degrading macromolecules and dysfunctional organelles, such as mitochondria and lysosomes [63]. Oxidative stress, starvation, hypoxia, inflammation, and infection are some examples of stress signals that can activate autophagy and result in an increase in autophagy [64, 65]. MSC homeostasis depends on autophagy for the quality control of proteins and organelles, which declines with aging. For example, Song and colleagues reported that short exposure (1 h) of BM-MSCs to H_2_ O_2_ dramatically elevates autophagic flux (2- to 4-fold), whereas 6h prolonged oxidative treatment reduces autophagy but enhances caspase-3 and caspase-6-associated apoptosis [66].

BM-MSCs from aged donors exhibit a significant reduction in autophagy than young BM-MSCs [67]. When used the autophagy inhibitor 3-methyladenine (3-MA) can induce young BM-MSCs to enter a relatively senescent state. Interestingly, the aging process of BM-MSCs can be reversed by the utilization of rapamycin [68]. Autophagy inhibits MSC senescence and promotes it by regulating rapamycin, optineurin, and p53 [69]. These diametrically opposed effects can be explained by the dual effects of autophagy regulation on MSC senescence. Optineurin (OPTN) is found to play a pivotal role in selective autophagy, coupling autophagy with bone metabolism. According to Liu and colleagues, OPTN has reduced expression in MSCs from aged mice, and this lower expression may contribute to bone loss through selective autophagy [70]. It has been shown that more than 85% of BM-MSCs showed up-regulation of p53 at passage 6. When down-regulation of the mammalian target of rapamycin (mTOR), aged BM-MSCs exhibited an increased expression of autophagy-related genes (LC3 and atg12) as compared to p2 BM-MSCs. During BM- MSC senescence, autophagy is important for maintaining MSCs by degrading large aggregates and damaged organelles. When p53 is knocked down, senescence is alleviated as well as autophagic activity is reduced, causing mTOR levels to increase. These results suggest that autophagy increases when BM-MSCs enter a replicative senescence state, and that p53 plays a key role in regulating autophagy during aging process [57]. In senescent MSCs, GATA Binding Protein 4 (GATA4) is a key regulator of senescence [71]. GATA4 activates transcription factors NF-κB and MCP-1 to cause SASP and induce MSC senescence, activate selective autophagy, and inhibit senescence by promoting degradation of senescence regulator GATA4 [72]. Hence, autophagy is responsible for clearing damaged proteins and organelles and maintaining and enhancing MSCs function during aging. Therefore, maintaining the ideal level of protein homeostasis may be a suitable strategy to enhance the effects of MSC transplantation therapy.

**Figure 2.**
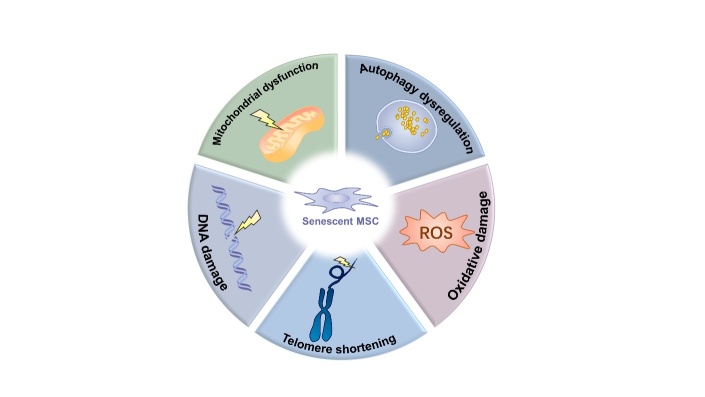
Molecular mechanisms of MSC senescence during aging. Several molecular mechanisms are associated with MSC senescence. DNA damage is a common underlying cause of MSC senescence in which the DNA damage response pathway is activated to block cell cycle progression and delay proliferative arrest. Telomeres gradually shorten with every cell division. Shortening of telomeres can induce cell cycle arrest and apoptosis of MSCs. Cellular senescence occurs when telomeres reach a critical length. The accumulation of dysfunctional mitochondria is associated with an increase in oxidative stress in senescent MSCs. Mitochondrial dysfunction results in an accumulation of ROS, which leads not only to MSC senescence on its own but also to their senescence by impairing mitophagy. ROS increases in aged MSCs, overproduction of endogenous ROS, and oxidative stress damage create a positive feedback loop. Autophagy is responsible for clearing damaged proteins and organelles and maintaining and enhancing MSCs function during aging. Autophagy dysregulation will promote MSC senescence. ROS, reactive oxygen species.

### 3.3 Metabolism dysfunction

Mitochondria is important for cellular metabolic homeostasis, signaling, differentiation, and aging. Mitochondrial dysfunction leads to increased mitochondrial fusion and decreased mitochondrial fission, decreased ATP production, decreased antioxidant capacity, increased ROS, and metabolic disturbances, and other pathological changes occurring in MSCs during aging [73]. When chronic inflammatory responses occur in aging, they will activate the Wnt/β-catenin pathway to inhibit mitophagy, so that damaged mitochondria accumulate in MSCs and impair differentiation [74]. Kim and colleagues compared with normal UC-MSCs, gestational diabetes mellitus (GDM) patients’ UC-MSCs showed decreased cell growth and earlier cellular senescence with accumulation of p16 and p53, mitochondrial dysfunction, and reduced expression of mitochondria function-regulating genes [75]. These findings demonstrated a strong relationship between mitochondrial dysfunction and MSC senesecence. Mitochondrial dysfunction results in an accumulation of ROS, which leads not only to MSC senescence on its own but also to their senescence by impairing mitophagy[76]. In MSCs, mitophagy is also vital to maintaining cellular homeostasis and preventing senescence. ROS accumulation attenuates mitophagy by down-regulating p38 and JNK phosphorylation and up-regulating the extracellular regulated protein kinases (ERK) phosphorylation, resulting in the induction of MSC senescence [77]. MSCs may be protected against oxidative stress by mitophagy, and impaired mitophagy leads to accumulation of ROS and dysfunctional mitochondria, which results in aging [78]. When reducing mitophagy levels and rendering cells unable to clear damaged mitochondria, downregulation of p53 can reverse the senescence of BM-MSCs [79]. Thus, interventions aimed at improving mitochondrial homeostasis in aged organisms may be able to have a significant impact on MSCs transplantation therapy.

**Figure 3.**
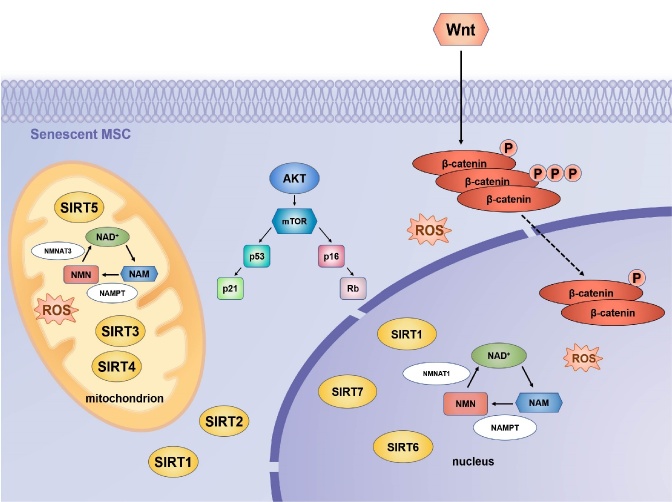
Signalling pathways and transcription factors involved in MSC senescence. Three major pathways contribute to the senescence of MSCs: Sirtuins/NAD+, AKT/mTOR, and Wnt/β-catenin and some transcription factors. The Sirtuin family of NAD-dependent protein deacetylases affects the functioning and metabolism of MSCs. SIRT1, SIRT6, and SIRT7 are found in the nucleus, SIRT2 is found in the cytoplasm, and SIRT3, SIRT4, and SIRT5 are found in the mitochondria. The knockout of Sirtuin induces the senescence of MSCs and inhibits their proliferation. ROS and other pro-senescence factors activate the p53-p21 and p16INK4A cell cycle arrest pathways. Consequently, retinoblastoma protein (Rb) is maintained in a hypophosphorylated state, which suppresses the expression of S-phase genes. AKT/mTOR signaling activates the p53/p21 and Rb/p16 pathways to block cell cycle progression and maintain an arrest in cell growth. Wnt/β-catenin signaling can lead to MSC senescence by promoting the production of intracellular ROS. Oxidative stress-associated β-catenin downregulation is involved in MSC senescence during aging. SRIT, Sirtuin; ROS, reactive oxygen species; mTOR, mammalian target of rapamycin; NAD+, oxidized nicotinamide adenine dinucleotide; NMN, nicotinamide mononucleotide; NAM, nicotinamide; Nampt, nicotinamide phosphoribosyltransferase; NAMPT, nicotinamide phosphoribosyltransferase; NMNAT, Nicotinamide mononucleotide adenylyltransferase.

Additionally, ROS plays a significant role in the fate of MSCs. Mild levels of ROS influence numerous signaling pathways that are involved in the proliferation and differentiation of MSCs [80]. However, during aging, ROS increases in aged MSCs, overproduction of endogenous ROS, and oxidative stress damage create a positive feedback loop[81]. Additionally, as MSCs age, oxidative stress and genomic stress (for example, oxidative stress-induced damage) may also affect signals and transcription factors. For example, the transcription factor nuclear factor erythroid 2-related factor 2 (Nrf2) promotes the expression of various target genes, such as NADPH quinone oxidoreductases 1 (NQO1) and heme oxygenases-1 (HO-1), these genes encode antioxidant mediators, promote the expression of antioxidant enzymes, and exert antioxidant activity [82]. The inhibition of the Nrf2/ARE signaling pathway results in diminished proliferation, colony formation, and osteogenic differentiation of BM-MSCs, as well as the accumulation of ROS, which reduces the stemness of aged BM-MSCs [83]. Activation of the Nrf2 pathway can reduce the expression of senescence markers in MSC and increase their antioxidant capacity, improving their senescence [84]. We can draw conclusions from the above studies that metabolism dysfunction caused by mitochondrial dysfunction and oxidative stress damage may lead to MSC senescence. Overall, it will be important to understand how metabolites and enzymes interact with each other.

### 3.4 Signaling pathways and transcription factors

Aging is a dynamic process during which overlapping but distinct molecular pathways are involved in cellular senescence at different stages. The regulation of signaling pathways and transcription factors play some crucial roles in the aging process of MSCs (Fig. 3).

#### 3.4.1 Sirtuins/NAD^+^ pathway

There is a fundamental connection between stem cell function and metabolic state via the silent information regulator proteins (Sirtuins) family of nicotinamide adenine dinucleotide (NAD)-dependent protein deacetylases [85]. SIRT1 is the earliest and most studied member of the sirtuins family. SIRT1 overexpression delayed MSC senescence by increasing telomerase activity, reducing DNA damage, and protecting MSCs from oxidative stress damage, while SIRT1 knockout induces cell senescence and inhibits cell proliferation [86]. Another member of sirtuins, SIRT3, is essential for restoring the differentiation capability of senescent MSCs [87]. Activation of antioxidant enzymes such as catalase (CAT) and manganese superoxide dismutase (MnSOD) may be positively regulated by SIRT3 through increasing forkhead box class O3a (FOXO3a) expression in the nucleus. It may also protect senescent MSCs from oxidative stress damage [88]. Ma and colleagues reported significant reductions in SIRT3 expression in senescent MSCs, which corresponded to reduced ROS and increased DNA damage. Replating SIRT3 could partially reverse senescence-associated phenotypic features in natural and prematurely senescent MSCs by alleviating ROS-induced injury and increasing superoxide dismutase 2(SOD2) expression and activity [89]. SIRT6 promotes osteogenic differentiation by interacting with BMP signaling in MSCs, and when SIRT6 was knocked out, the proliferation and migration of senescent BM-MSCs were reduced [90, 91]. What’s more, Bi and colleagues found that SIRT7 expression was reduced, and SIRT7 deficiency accelerated MSC senescence[92]. Sirtuin activity and NAD^+^ levels both decline during aging. When the intracellular level of NAD^+^ was increased, SIRT1 activity was enhanced, partially restoring mitochondrial fitness and reversing the senescence of MSCs[93]. Finally, as the rate-limiting enzyme of NAD^+^, nicotinamide phosphoribosyltransferase (NAMPT) can restore aging defects in MSCs by affecting NAD^+^ level and Sirt1 activity directly [94, 95]. Nicotinamide mononucleotide (NMN), a key natural NAD^+^ intermediate, promoted MSC self-renewal in aged mice through an upregulation of SIRT1 that augmented osteogenesis and reduced adipogenesis[96]. In the present study, senescent MSCs were found to have a reduced NAD^+^ content, a reduced SIRT3 expression level, and mitochondrial dysfunction. NMN supplementation increased intracellular NAD^+^ levels, NAD^+^/NADH ratios, SIRT3 expression, as well as mitochondrial function and rescued senescent MSCs [97]. These studies suggest that working at the level of sirtuins may be one way of combating aging. Consequently, MSC senescence was associated with a reduction in sirtuins, and sirtuins overexpression reversed some senescence-related phenotypes. It may be possible to combat MSC senescence by acting at the sirtuins protein level.

#### 3.4.2 AKT/mTOR signaling pathway

Senescent cells can activate the mTOR pathway, which is critical for regulating autophagy and cellular senescence as well as controlling the production of SASP[98]. mTOR is composed of the mammalian target of rapamycin complex 1 (mTORC1) and the mammalian target of rapamycin complex 2 (mTORC2). mTORC1 primarily controls cell growth, while mTORC2 participates in the control of cell survival and proliferation. BM-MSC proliferation is negatively regulated by TSC1, a tumor suppressor that inhibits cell growth via the negative regulation of mTORC1[99]. The AKT/mTOR signaling pathway plays a key role in MSC senescence, studies have shown that mTOR signaling is activated in aged BM-MSCs [100]. mTOR inhibition resulted in an increase in the number of SA-β-gal positive cells and autophagy protein levels [101]. Using phosphatidylinositol Akt/mTOR inhibitors, Gharibi and colleagues demonstrated that MSCs with early passage morphology exhibit high clonogenic frequencies and enhanced proliferative capacities [102]. Zhang and colleagues demonstrate that CoQ10 can attenuate MSC senescence by inhibiting ROS generation, and the Akt/mTOR signaling may play a critical role in MSC senescence inhibited by CoQ10 [103]. In mice treated with rapamycin, a potent SASP suppressor, rapamycin significantly increased lifespan [104]. Therefore, inhibiting mTOR signaling or restoring Akt kinase activity may be a promising therapeutic target for protecting aged MSCs.

#### 3.4.3 Wnt/β-catenin signaling pathway

Wnt/β-catenin signaling is the key regulator of self-renewal and differentiation in MSCs [105]. The results demonstrated that Wnt/β-catenin signaling can lead to MSC senescence by promoting the production of intracellular ROS [106]. It was determined that after treating BM-MSCs with Wnt/β-catenin signaling pathway inhibitors for 48 h, A significant decrease in p53 expression and a reversal of senescence characteristics were noted in BM-MSCs, indicating that this signaling pathway may play a significant role in BM-MSC senescence [107]. Xia and colleagues found that the Wnt/β-catenin signaling pathway appeared to be inhibited in aged MSCs from aged (18-month-old) mice[108]. Researchers have discovered that overexpression of miR-1292 significantly accelerates the aging process and inhibits osteogenesis. Analysis results indicate that miR-1292 modulates AD-MSC senescence status and osteogenic differentiation primarily through Wnt/β-catenin signaling [109]. Signaling protein WNT3A counteracts the induction of paracrine senescence in MSCs, WNT signal pathway interrupts this cascade by repressing cytokines that mediate this induction of senescent MSCs [110]. WNT5A as the FZD5 ligand, treatment of MSCs with WNT5A promoted their proliferation [111]. In a word, Wnt/β-catenin signaling might be one of the main contributors to MSC senescence. Excessive activation of the wnt/β-catenin pathway may be one of the primary factors leading to MSC senescence. Identifying how different mechanisms and pathways impact MSCs and determining how these pathways interact with external stimuli to modulate MSC aging, and rejuvenation will be important.

**Figure 4.**
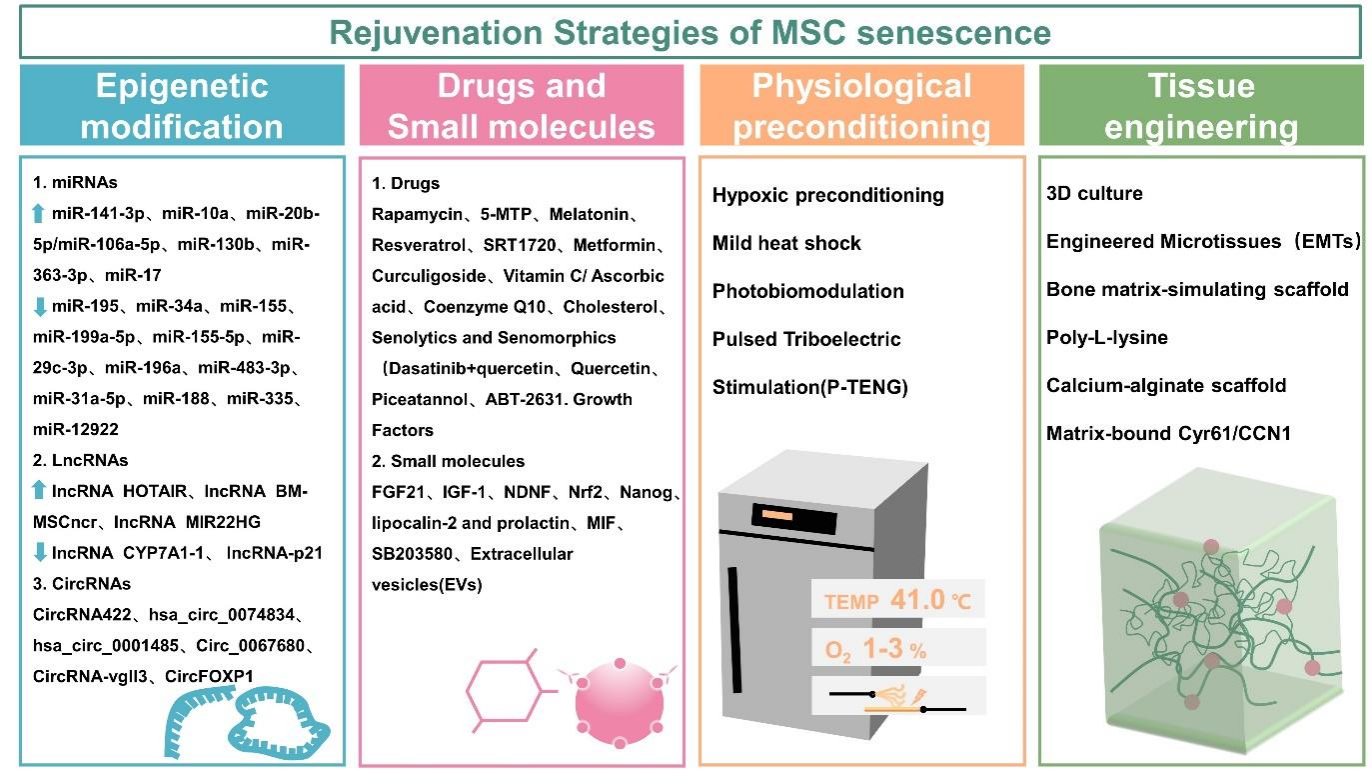
Rejuvenation strategies of MSC senescence. The main rejuvenation strategies for MSC senescence include epigenetic modification, primarily by regulating noncoding RNAs (miRNAs, lnRNAs, circRNAs); drugs and small molecules; physiological preconditioning (by changing oxygen content and temperature during cell culture, etc.); tissue engineering (by modifying cells nurturing environment). miRNAs, microRNAs; lncRNAs, long noncoding RNAs; circRNAs, circular RNAs; 5-MTP, 5-methoxytryptophan; FGF, fibroblast growth factor; IGF, insulin-like growth factor; NDNF, neuron-derived neurotrophic factor; MIF, migration inhibitory factor.

## 4. Rejuvenation Strategies of MSC senescence

The MSC niche is defined as a specialized local microenvironment composed of MSCs, non-stem support cells, protein, soluble factors, and the extracellular matrix (ECM) that supports multilineage differentiation and robust self-renewal of MSCs [112]. For example, the bone marrow is resident to BM-MSCs, which are localized in the MSC niche regulating MSCs quiescence, self-renewal, and differentiation [113]. In the niche, MSCs are normally silent, preventing the accumulation of genetic damage [114].

The contents of this review reveal that MSC senescence has a complex mechanism, Thus, a variety of approaches are necessary to reverse or prevent senescence as well as to enhance the clinical application of MSCs. Aging is reversible, and many scientists are proposing strategies to counteract senescence and improve the health and therapeutic potential of MSCs. We aim to discuss some ways to therapeutically target the cellular processes underlying aging in order to shorten the period of functional decline associated with aging. In this way, multiple diseases associated with aging could be prevented, delayed, or alleviated simultaneously. As a consequence of some interventions, MSCs may be able to delay senescence and restore youthful properties by modulating MSC niches or directly impacting MSCs states. In this review, we examine how a variety of rejuvenation strategies affect tissue MSCs. The following are some interventions that have been reported to affect the function of aged MSCs (Fig. 4).

### 4.1 Noncoding RNAs

An interesting intervention towards rejuvenation is epigenetic reprogramming. Epigenetic reprogramming includes epigenetic modifications, in addition to coding genes, also noncoding RNAs (miRNAs, lnRNAs, circRNAs) that regulate gene expression. Modulating noncoding RNAs is an approach to counteracting senescence by targeting molecules directly or indirectly involved in senescence (Table 1).

**Table 1:** Non-coding RNAs regulating MSC senescence.

Non-coding RNAs	MSC type	Target	Effect	Ref.
Inhibition of miR-195	BM-MSC	Target to 3'-untranslated region of TERT gene	Telomere relengthening	[115]
Overexpression of miR-141-3p	UCB-MSC	Target to ZMPSTE24	Upregulate prelamin A	[116]
Inhibition of miR-34a	BM-MSC	Target to TP53	Reduce ROS	[117]
Inhibition of miR-155	BM-MSC	Target to Bcl-2 associated BAG5	Enhance mitophagy	[118]
Inhibition of miR-199a-5p	AD-MSC	Target to the SIRT1/AMPK signaling pathway	Promotes autophagy and ameliorates MSC senescence	[119]
Inhibition of miR-155-5p	BM-MSC	Target to the AMPK signaling pathway	Rejuvenate aged MSC	[28]
Overexpression of miR-10a	BM-MSC	Target to the BAX/Bcl-2 pathway	Rejuvenate aged MSC and improved cardiac function	[120]
Inhibition of miR-29c-3p	BM-MSC	Target to CNOT6 through p53-p21 and p16-pRB pathways	Rejuvenate osteogenic differentiation capacity of senescent MSC	[121]
Overexpression of miR-20b-5p/miR-106a-5p	WJ-MSC	Target to the p21/CDK/E2F1 pathway	Promote cell proliferation, G1/S transition and DNA synthesis of MSC	[122]
Inhibition of miR-196a	BM-MSC	Target to HOXB7	Improve proliferation and osteogenesis	[123]
Overexpression of miR-130b	BM-MSC	Target to the ERK/FOXM1 pathway	Against HG-induced MSC senescence	[124]
Inhibition of miR-483-3p	AD-MSC	Target to insulin-like growth factor-1 (IGF1)	Reduce cellular senescence	[125]
Overexpression of miR-363-3p	BM-MSC	Target to tumor necrosis factor receptor-associated factor 3 (TRAF3)	Promote BM-MSC osteogenic differentiation and suppresses osteogenic differentiation and senescence	[126]
Overexpression of miR-17	BM-MSC	Target to the p53/miR-17/Smurf1 pathway	Rescue the osteogenic differentiation of aged BM-MSC	[127]
Inhibition of miR-31a-5p	BM-MSC	Target to the SATB2/E2F2/RhoA pathway	Modulate osteoclastic differentiation of aged MSC	[128]
Inhibition of miR-188	BM-MSC	Target to HDAC9 and RICTOR	Stimulate bone formation and decrease marrow fat accumulation in aged mice	[52]
Inhibition of miR-335	BM-MSC	Target to AP-1	Rejuvenate aged mesenchymal stem cells	[129]
Inhibition of miR-1292	AD-MSC	Target to FZD4	Regulate Senescence and Osteogenic Differentiation	[109]
Downregulation of lncRNA CYP7A1-1	BM-MSC	Target to SYNE1	Rejuvenate aged BM-MSC	[131]
Overexpression of lncRNA HOTAIR	BM-MSC	Target to senescence-associated DNAm.	Impact on proliferation and differentiation of MSC	[132]
Inhibition of lncRNA p21	BM-MSC	Target to the Wnt/β-catenin signaling pathway	Rejuvenate aged MSC	[133]
Overexpression of lncRNA Bmncr	BM-MSC	Target to the BMP2 pathway	Alleviate bone loss and bone marrow fat accumulation	[134]
Overexpression of lncRNA miR22HG	BM-MSC	Target to the PTEN/AKT pathway	Enhanced osteogenic differentiation of aged MSCs	[135]
CircRNA422	BM-MSC	Target to the SP7/LRP5 axis	Enhanced osteogenic differentiation of aged BM-MSCs	[137]
CircRNA hsa_circ_0074834	BM-MSC	Target to the expression of ZEB1 and VEGF	Enhanced osteogenic differentiation of aged BM-MSCs	[138]
CircRNA hsa_circ_0001485	BM-MSC	Target to the TGFβ-BMP signaling pathway	Enhanced osteogenic differentiation of aged BM-MSCs	[139]
Circ_0067680	BM-MSC	Target to the Wnt/β-catenin signaling pathway	Enhanced osteogenic differentiation of aged BM-MSCs	[140]
CircRNA-vgll3	AD-MSC	Target to the circRNA-vgll3/miR-326-5p/ Itga5 pathway	Enhances the osteogenic differentiation of aged AD-MSCs	[141]
CircFOXP1	AD-MSC	Target to FOXP1 gene	Bind to miR-33a-5p promotes osteogenic differentiation of aged AD-MSCs	[142]

#### 4.1.1 miRNAs

miRNAs are small, about 20 nucleotide long molecules that exist both inside and outside of the cell. This molecule interferes with transcription through a variety of mechanisms, the most common of which is interaction with the target gene RNA. The 3'UTR sequence of mRNA is combined to silence or degrade the mRNA. They play a biological role by interfering with RNA replication. Several miRNAs have been validated for reversing MSC senescence by extending telomere length, regulating autophagy, removing excess ROS, and improving the biochemical property. Researchers have found that miR-195 directly targeted the 3'-untranslated region of the TERT gene and the knockdown of miR-195 significantly increased TERT expression in aged MSCs. Strikingly, miR-195 inhibition significantly induced telomere lengthening in senescent MSCs [115]. By suppressing ZMPSTE24 expression, miR-141-3p acts as a negative regulator of cellular senescence, which leads to increased accumulation of prelamin A with increasing senescence [116]. As a TP53-targeted miRNA, miR-34a modulates cell senescence and its overexpression may exacerbate MSC senescence, while its inhibition may reduce ROS levels in aged MSCs [117]. miR-155 expression was increased in aged MSCs, as well as the impairment of mitophagy in MSCs[118]. As reported by Shi and colleagues, activating the SIRT1/AMPK pathway by inhibiting miR-199a-5p promotes autophagy and ameliorates MSC senescence [119]. In MSCs from aged donors, miR-155-5p expression was significantly higher than that from young donors. Downregulation of miR-155-5p decreased the senescence of MSC [28]. By inhibiting the BAX/Bcl-2 pathway, miR-10a overexpression rejuvenated senescent BM-MSCs and improved cardiac function and angiogenesis in injured mouse hearts[120]. The miR-29c-3p level increased significantly during the aging process of MSCs, whereas miR-29c-3p downregulation inhibited osteogenic differentiation and induced changes in senescence markers when over-expressed [121]. H_2_ O_2_ treatment hampered G1/S transition in hUC-MSCs and suppressed DNA synthesis, whereas miR-20b-5p/miR-106a-5p overexpression prevented growth arrest and promoted G1/S transition[122]. The up-regulation of miR-196a expression is also correlated with the impairment of osteogenesis in aged MSCs. The forced expression of HOXB7, a target gene for miR-196a, was associated with improved proliferation and osteogenesis, as well as a reduction during aging[123]. Up-regulation of miR-130b protects MSCs from senescence induced by high glucose levels, possibly through activation of the ER/FOXM1 pathway [124].

In addition, miRNAs can impact the property of aged MSCs. Knocking down miR-483-3p retarded adipogenic differentiation of AD-MSCs and reduced cellular senescence [125]. Overexpression of miR-363-3p attenuated the effects of TRAF3 on BM-MSCs adipogenic differentiation, osteogenic differentiation, and senescence [126]. The protein smurf1 is a direct target gene of miR-17. It has been found that overexpression of miR-17 can improve the osteogenic ability of aged MSCs [127]. miR-31a-5p is also known to promote osteoclast differentiation via the RhoA pathway. Aged rats were also protected against bone loss and osteoclastic activity by inhibition of miR-31a-5p [128]. In aged mice, miR-188 is highly expressed in MSCs, and miR-188 inhibition increases bone formation and reduces bone marrow fat accumulation[52]. Forced expression of miR-335 can result in early senescence-like defects related to AP-1 activity, while inhibition of miR-335 expression reverses this effect on MSCs function [129]. By knocking down miR-1292, Fan and coauthors were able to reduce senescence in AD-MSCs and enhance osteogenic differentiation through Wnt/β-catenin signaling [109].

#### 4.1.2 LncRNAs

A long non-coding RNA (LncRNA) is a non-coding RNA with a length of more than 200 base pairs. Although lncRNAs are not involved in making antisense, intronic or intergenic transcripts, the LncRNAs’ effect is at the transcriptional and post-transcriptional level, and they can influence translation while acting as“sponges” for miRs and proteins. Recent studies have found that a number of lncRNAs, referred to as senescence-associated lncRNAs (SAL-RNAs), are involved in regulating senescence [130]. Dong and colleagues came to the conclusion that lnc-CYP7A1-1 changed with aging. In senescent BM-MSCs, downregulation of lnc-CYP7A1-1 led to MSC regeneration, however inhibition of SYNE1 reduced this effect[131]. Kalwa and colleagues focused on senescence-associated changes of lncRNA HOTAIR in gene expression and DNA-methylation (DNAm) in MSCs. Their results demonstrated that HOTAIR impacts on differentiation and proliferation of old MSCs and that it is associated with senescence-associated DNAm [132]. As compared to MSCs isolated from young mice, Xia and colleagues found that aged MSCs showed reduced proliferation and paracrine signaling, as well as an increased oxidative stress level and expression of the lincRNA-p21 gene. Silencing of lincRNA-p21 by small interfering RNA (siRNA) improved aged MSCs growth and paracrine function, as well as decreased oxidative stress [133]. Li and colleagues identified lncRNA Bmncr, which regulated the fate of BM-MSCs during aging. By maintaining the extracellular matrix protein fibronectin (FMOD) and activating the BMP2 pathway, Bmncr regulates the osteogenic niche of BM-MSCs. Overexpression of Bmncr alleviated bone loss and fat accumulation in bone marrow[134]. Interestingly, it has been demonstrated that MIR22HG, a long non-coding RNA, promotes osteogenic differentiation of hBM-MSCs via the PTEN/AKT pathway [135].

**Table 2:** Preconditioning with drugs and small molecules regulating MSC senescence.

Biomolecules /Chemicals	MSC type	Target	Effect	Ref.
Rapamycin	BM-MSC	1. Target to autophagy 2. Target to the Akt/mTOR pathway	1. Restore the bone loss in aged MSCs 2. Prevents the development of an age-related phenotype and maintains MSC morphology of early passage cells	[102, 143]
5-MTP	BM-MSC	1. Target to FoxO3a and mTOR 2. Target to mitochondrial function and reducing ROS generation	Rescue MSCs from Senescence	[144, 145]
Melatonin	AD-MSC/ BM-MSC	1. Target to mitochondrial function and mitophagy 2. Target to ROS and p53/ERK/p38 3.Target to PrPC and mitochondrial function 4. Target to the SIRT1-dependent pathway	Rescue MSC from senescence	[146-149]
Resveratrol	AD-MSC/ UC-MSC/ BM-MSC	1. Target to the p-Akt expression 2. Target to the SIRT1 3. Target to the mitochondrial autonomous gene transcription	1. Enhance AD-MSCs viability 2. Promote cell viability and proliferation, mitigate senescence of UD-MSCs 3. Improve mitochondrial functional recovery and osteogenic differentiation of senescent BM-MSCs	[150-153]
SRT1720	BM-MSC	Target to the SIRT1 and FAIM	Improve survival of aged MSCs	[154]
Metformin	AD-MSC	1. Target to the SOD 2. Target to NF-κB and SASP	1. Reduce expression of senescence markers and decreased amount of ROS 2. Inhibit MSC senescence and DNA damage	[155, 156]
Curculigoside	BM-MSC	Target to the TAZ expression	Increase osteogenesis and decrease adipogenesis of aged BM-MSCs	[157]
Vitamin C/ Ascorbic acid	SCB-MSC/ BM-MSC	1. Target to the prelamin A expression 2. Target to ROS and the AKT/mTOR signaling pathway	Reverse aging in MSCs	[100, 158]
Coenzyme Q10	BM-MSC	Target to the Akt/mTOR signaling pathway	Inhibit MSC aging	[103]
Cholesterol	BM-MSC	Target to the cell cycle, autophagy, and ROS/p53/p21 signaling pathways	Rejuvenate MSC senescence	[159]
Senolytics and Senomorphic	BM-MSC/ synovial MSC	Target to remove senescent cells	1. Improve proliferation and osteogenic capacity of aged BM-MSCs 2. Promote the stemness properties of MSC	[161-164]
FGF21	BM-MSC	Target to the AMPK signaling pathway	Reduce ROS and inhibite MSC senescence	[73]
IGF-1	BM-MSC	Target to the osteogenic potential	Increase the proliferation rate and osteogenic potential of agd BM-MSCs	[165]
NDNF	BM-MSC	Target to the p-Akt pathway	Rejuvenate aged MSCs	[166]
Nrf2	UC-MSC	Target to IDO-1 expression	Regulation of the immunosuppressive properties and cellular senescence of MSCs	[167]
Nanog	BM-MSC	Target to the TGF-β pathway	Enhanced the proliferation rate and clonogenic capacity of aged BM-MSCs	[168]
Lipocalin-2 and prolactin	BM-MSC	Target to mimic the bone marrow niche	Delay cellular senescence of BM-MSCs	[169]
MIF	BM-MSC	Target to autophagy	Induce MSC senescence	[170, 171]
SB203580	ED-MSC	Target to the p38MAPK pathway	Recover senescence phenotype	[172]
Extracellular vesicles (EVs)	BM-MSC	Target to the IGF1/PI3K/AKT pathway transferring PCNA the miR-183-5p target heme oxygenase-1 (Hmox1)	improve the function and stemness of MSCs	[174-178]

#### 4.1.3 CircRNAs

It has been found that a number of endogenous circular RNAs exist in various tissues and that circular RNAs play a role in determining the fate of MSCs in recent studies [136]. For example, circRNA422, circ_0067680, hsa_ circ_0074834, and hsa_circ_0001485 significantly enhanced osteogenic differentiation of BM-MSCs[137-140]. Additionally, circRNA-vgll3, originating from the vgll3 locus, enhances osteogenic differentiation in AD-MSCs [141]. Shen and colleagues reported that circFOXP1 was significantly down-regulated in osteoporosis patients' bone tissue and that the miRNA sponge targeted miR-33a-5p in order to increase the expression of FOXP1. By targeting FOXP1, circFOXP1 binds to miR-33a-5p to promote osteogenic differentiation of hAD-MSCs [142].

An important factor regulating the function of MSCs is aging, which is also associated with changes in the expression of noncoding RNAs. Despite this, most noncoding RNAs are not fully understood in terms of their biological functions. Focus on the epigenetic reprogramming of BM-MSCs during aging and may represent the MSCs therapeutic strategy.

### 4.2 Drugs and Small molecules

MSCs are affected by other components of niche, and distant tissues and systems may transmit soluble factors and extracellular vesicles through the circulatory system. Studying the composition and properties of MSC niches will enable us to better understand how MSCs are regulated by these niches. Creating a culture microenvironment that mimics the MSC niche would be beneficial for maintaining the MSC property effectively in culture. Various rejuvenation strategies have been reported to impact the function of senescent MSCs through the use of biomolecules and chemicals. Involving preconditioning interventions (the use of drugs, cytokines, physical factors, etc.) to reverse MSC senescence by modulating senescence-related signaling pathways, improving damaged cell function, and removing senescent cells (Table 2 and 3).

#### 4.2.1 Drugs

SASP factors play a role in the transmission of inflammatory responses and contribute to various age-related pathological conditions and trigger senescence in neighboring cells. Some SASP inhibitors, such as rapamycin, melatonin, resveratrol, metformin, and sirtuin activators, have been shown to slow age-related tissue degeneration and prolong lifespan. For example, rapamycin delays aging-related diseases, as well as prevents the development of MSC senescence by increasing cloning frequency and proliferation via the Akt/mTOR pathway [102, 143]. 5-Methoxytryptophan (5-MTP) and melatonin reverse MSC senescence by protecting mitochondrial integrity and function and reducing ROS production, protecting aged MSCs [144, 145]. Interestingly, pretreatment MSCs with melatonin prevents replicative senescence by activating heat shock protein 1L (HSPA1L) by restoring mitochondrial dynamics [146]. A study conducted by Han and colleagues found that melatonin administration prevents excessive senescence in MSCs by upregulating PRPC and enhancing mitochondrial function [147]. Additionally, melatonin inhibits ROS accumulation, activates p53/ERK/p38, and protects MSCs from premature senescence through Sirtuin 1 signaling pathway [148, 149]. As the natural plant polyphenol, Recent studies have shown that resveratrol (RESV) activates SIRT1, thereby exerting anti-aging effects [150]. For example, AD-MSC pretreatment with resveratrol enhances AD-MSCs viability [151]. By inhibiting the expression of p53 and p16, co-culturing UC-MSCs and RESV increased cell proliferation and induced SIRT1 and proliferating cell nuclear antigen (PCNA) expression [152]. The study by Lv and colleagues indicate that chronic intermittent application of RESV improves osteogenic differentiation and increases the metabolism of aged MSCs by promoting mitochondrial-autonomous gene transcription [153]. What’s more, pretreatment of aged MSCs with a SIRT1 activator, SRT1720, confers an improvement in the survival of aged MSCs by activating SIRT1 and upregulating FAIM [154]. In AD-MSCs treated with metformin, senescence-related markers and ROS were reduced, proliferation potential was increased, osteogenic differentiation potential decreased, and adipogenic differentiation potential increased [155]. Kim and colleagues analyzed the effects of metformin on the aged AD-MSCs in chronic kidney disease (CKD) patients. Compared with control MSCs, MSCs in patients with CKD exhibited decreased proliferation, accelerated aging, and increased DNA damage. These changes were significantly attenuated after metformin pretreatment [156]. Several common drugs that have been used clinically have also been shown to reverse the senescence of MSCs. For example, Curculigoside (CCG) increases the expression of TAZ in aged BM-MSCs, which increases osteogenesis and decreases adipogenesis [157]. Vitamin C reverses MSC senescence by inhibiting prelamin A expression in subchondral bone-derived MSCs (SCB-MSCs), reducing the secretion of inflammatory factors, inhibiting the cell cycle, and inhibiting ROS generation and the activation of the AKT/mTOR signaling pathway, which impair MSCs senescence [100, 158]. A study found that Coenzyme Q10 inhibited D-gal-induced aging of MSCs via Akt/mTOR pathway [103]. Cholesterol is involved in many functional processes such as cell proliferation and differentiation. Cholesterol appears to play a functional role in BM-MSC senescence by regulating the cell cycle, autophagy, and ROS/p53/p21 signaling pathways [159].

**Table 3:** Preconditioning with Physiological factors and tissue engineering regulating MSC senescence.

Physiological factors/ tissue engineering	MSC type	Target	Effect	Ref.
Hypoxic preconditioning	OM-MSC/ BM-MSC/AD-MSC	1. Target to autophagy 2. Target to VEGF 3. Target to oxidative stress, DNA damage, telomere shortening and chromosomal aberrations.	1. Delay MSC senescence 2. Enhance neuroprotective effects of aged MSCs 3. significantly increases lifespan of MSC	[180-182, 184]
Mild heat shock	AD-MSC	Target to HSC70	Activate proliferation of aged MSC	[185]
Photobiomodulation	BM-MSC	Target to mitochondrial functionality	Rejuvenate aged MSC	[186]
Pulsed Triboelectric Stimulation (P-TENG)	BM-MSC	Target to MDM2-dependent p53 degradation	Rejuvenate senescent BM-MSC	[187]
Three- dimensional culture	AD-MSC	Target to telomere length and telomerase activity	MSC senescence-related changes improved	[189]
Engineered Microtissues (EMTs)	BM-MSC	Target to TGF-β3	Restore the Chondrogenic Potential of Aged MSC	[190]
Bone matrix-simulating scaffold	UC-MSC	Target to compatibility and bioactivity	Alleviate replicative senescence of MSC	[191]
Poly-L-lysine	BM-MSC	Target to the functionality and stemness of MSC	Prevent Senescence and augment Growth in Culturing MSC	[192]
Calcium-alginate scaffolds	BM-MSC	Target to bone formation	Increase the proliferation rate and osteogenic potential of aging BM-MSC	[165]
Matrix-bound Cyr61/CCN1	BM-MSC	Target to the matricellular protein, Cyr61/CCN1	Retente BM-MSCs’ proliferation and growth factor responsiveness	[193]

Two classes of therapeutic drugs, senolytics, and senomorphics, have been reported to attenuate aging-related pathological phenotypes in mice, and are currently being evaluated in humans. In senescent cells, Senolytics target senescent cells by inducing apoptosis, whereas senomorphics inhibit SASP that causes pro-inflammatory paracrine signaling and tissue damage [160]. Thus, they may delay the degeneration associated with aging. Zhang and colleagues demonstrated that Quercetin was used to clean senescent BM-MSCs and improves the proliferation and osteogenesis of BM-MSCs as well as inhibits the adipogenesis of BM-MSCs [161]. Zhou and colleagues employed a senolytic cocktail (dasatinib+quercetin; D + Q) to improve the proliferation and osteogenic capacity of aged BM-MSCs both in vitro and in vivo [162]. As a senolytic, piceatannol promoted the recovery of cell proliferation and the stemness property of MSCs by affecting p53-independent senescence [163]. In a study conducted by Miura and colleagues, senolytic drugs such as ABT-263 were shown to improve the biological function of synovial-derived MSCs through the removal of senescent cells [164]. However, the potentially harmful effects of killing or altering senescent cells in organs require further investigation. In order to accurately predict drug efficacy, studies involving senolytics and senomorphics must compare disease outcomes across treatment durations.

#### 4.2.2 Small molecules

As previously mentioned, therapeutic strategies targeting soluble factors and downstream effectors have been tested in aged MSCs.

(1) Growth factors: An alternative strategy to rejuvenate MSC senescence is growth factors pretreatment. Numerous growth factors have also been demonstrated to play an important role in rejuvenating MSCs that have reached senescence. A study indicated that when MSCs expanded in vitro, their expression of fibroblast growth factor 21 (FGF21) decreased, and overexpression of FGF21 reduced MSC senescence through the AMPK signaling pathway. MSC quality and quantity may be improved by targeting FGF21 [73]. Growth factor insulin-like growth factor 1 (IGF-1) plays a role in bone formation. When aged BM-MSCs were overexpressed with IGF-1, their proliferation rate increased, and their osteogenic potential was enhanced [165]. Overexpression of neuron-derived neurotrophic factor (NDNF) in aged BM-MSCs increased cell proliferation, survival, and angiogenesis in vitro by activating the p-Akt pathway [166]. In the elderly, NDNF may be able to rejuvenate aged MSCs and improve their ability to repair damaged hearts.
(2) Transcription factor: Treatment of MSCs with the transcription factor frizzled 5 (FZD5) ligand WNT5A promoted their proliferation. When FZD5 knockdown, MSCs exhibited markedly attenuated proliferation and differentiation ability [111]. As a transcription factor that responds to oxidative stress, Nrf2 was found to be down-regulated in long-term cultured UC-MSCs. Further knockdown of Nrf2 in UC-MSCs induced senescence, decreased proliferation, and regulated the immuno-suppressive properties of UC-MSCs [167]. Han and colleagues show that the forced expression of Nanog reversed the myogenic differentiation potential and restored the contractile function of adult BM-MSCs to a similar level as that of neonatal BM-MSCs [168].
(3) Paracrine factor: Studies of paracrine factor have revealed that soluble factors from MSC niches can impact aged MSC function. The bone marrow contains soluble factors such as lipocalin-2 and prolactin, which are key factors regulating the activity of BM-MSCs. When BM-MSCs are treated with lipocalin-2 and prolactin, the cell is delayed from entering senescence and is primed for osteogenesis and chondrogenesis [169]. In a recent study, it was found aged MSCs produced a lower level of macrophage migration inhibitory factor (MIF), whereas overexpression of MIF rejuvenated aged MSCs through the activation of autophagy [170]. MIF treatment also enhanced the growth, paracrine function, and survival of senescent MSCs [171]. SB203580 can inhibit the activity of p38^MAPK^ and inhibit the subsequent activation of MAPKAP Kinase-2 and MAPKAP Kinase-3. H_2_ O_2_ -induced senescence of endometrium-derived MSCs treatment with SB203580 was sufficient to recover partially senescence phenotype, block the ROS elevation, decrease the mitochondrial function, and finally rescue proliferation [172]. Extracellular vesicles (EVs), such as microvesicles and exosomes, are nanosized vesicles that are secreted by most cells. In addition to containing proteins, lipids, and RNAs of the source cell, EVs are reported to transmit these bioactive components from one cell to another [173]. In this way, EVs rejuvenate aged MSC by delivering their components. For example, human embryonic stem cell-derived small extracellular vesicles (hESC-sEVs) can rejuvenate aged BM-MSCs, thereby preventing age-related bone loss[174]. Zhang and colleagues identified that the IGF1/PI3K/AKT pathway mediated the anti-aging effects of ESC-EVs on MSCs [175]. Interestingly, EVs from non-aged MSCs were found to be beneficial for aged MSCs and improve the function and stemness of MSCs to delay the aging process [176]. For example, it has been shown that UC-MSCs-EVs rejuvenate adult BM-MSCs in part by transferring PCNA into recipient adult BM-MSCs [49]. Despite the lack of extensive research in this area, it represents an important area of research. Researchers recently reported that miR-183-5p increases with age in EVs isolated from BM-MSCs. The miR-183 cluster (miR-96/-182/-183) is highly expressed in old EVs. Also, they report that young BM-MSCs take up old bone marrow-derived EVs and inhibit osteogenic differentiation. Furthermore, transfection of miR-183-5p mimics reduced cell proliferation, Hmox1 protein levels, and increased senescence of BM-MSCs [177]. Interestingly, a study found that injecting BM-MSCs from a young mouse prolonged their life span and health span while injecting conditioned media (CM) from BM-MSCs from young mice rescued the function of aged MSCs and senescent fibroblasts [178]. As a result of these novel findings, EVs appear to be one of the most important factors released by young, functional MSCs to rescue senescence. In addition, a study indicated that culture on ECM, produced by BM-MSCs from young donors, improved the quantity and quality of aged BM-MSCs [179].

Comparatively to viral-based gene delivery methods, small molecules are non-immunogenic, cost-effective, and have associated protocols that are easy to standardize. Nevertheless, improvements are required in order to extend the duration of small molecule action and to precisely control the site at which they function in vivo in order to maximize reprogramming efficacy while minimizing side effects. Further research is necessary to determine how compounds and metabolites contribute to the function of MSCs during aging.

### 4.3 Physiological preconditioning

#### 4.3.1 Hypoxic preconditioning

In vitro culture, MSCs are typically cultured in an incubator with 21% oxygen, which is higher than the oxygen concentration in the MSC niche. Excessive oxygen increases oxidative stress and the activation of related signaling pathways, which ultimately results in cellular senescence. Consequently, hypoxia preconditioning of MSCs and HIF1-induced metabolic switch are key physiological processes involved in limiting oxidative stress, DNA damage, telomere shortening, and chromosomal aberrations [180]. For example, He and colleagues used next-generation sequencing to analyze both lncRNAs and mRNAs expression profiles of hOM-MSCs under hypoxia (3%) and normoxia preconditioning. Based on the enrichment analysis, it was discovered that differentially expressed genes of hOM-MSCs were primarily involved in the regulation of the cell cycle, secretion of cytokines. In addition, hypoxic conditions promoted cell proliferation of hOM-MSCs and inhibited cell apoptosis [181]. By upregulating miR-326/PTBP1/PI3K-mediated autophagy, the study by Liu and colleagues indicated that hypoxic preconditioning delayed the senescence of hOM-MSCs and enhanced their therapeutic effects in ICH [182]. The MSCs from patients with atherosclerotic renal artery stenosis (ARAS) display impaired function, senescence, and DNA damage. In a study by Isik and colleagues, hypoxic preconditioning mitigated MSC senescence in swine ARAS-MSCs. Therefore, hypoxic preconditioning of MSCs may be considered for improving autologous cell therapy in patients with nephropathy [183]. What’s more, a hypoxic preconditioning procedure enhances the protective effects of aged hBM-MSCs against cerebral ischemia in vitro [184]. These findings showed senescent MSCs were more effective in rejuvenation under hypoxic preconditioning.

#### 4.3.2 Others preconditioning

The regulation of temperature also plays a key role in reversing MSC senescence. For example, a number of heat shock proteins, including the major stress protein HSP70, are involved in maintaining intracellular homeostasis and preventing protein damage, Andreeva and colleagues reported that mild heat shock (41 °C, 60 min) increased HSC70 levels and significantly activate proliferation in aged hMSCs [185]. Eroglu and colleagues reported that a near-infrared photobiomodulation (PBM) treatment delivering 3 J/cm^2^ is the most effective modality for improving mitochondrial functionality and aging markers. On senescent MSCs, PBM has a lasting rejuvenating effect[186]. In a study, a pulsed triboelectric nanogenerator (P-TENG) was constructed based on triboelectric stimulation. P-TENG revitalizes aged BM-MSCs through the promotion of MDM2-dependent p53 degradation, enhancing the proliferation and increasing their pluripotency and differentiation potential of aged BM-MSCs [187].

### 4.4 Tissue engineering

When MSCs are cultivated in vitro the senescence of these cells can also be reversed by altering their culture environment. Compared to traditional adherent culture, 3D culture, as a new MSCs culture process, has the advantage of greater culture efficiency [188]. For example, comparing the aging-related changes in MSCs isolated from human adipose tissue in two-and three-dimensional (3D) cultures, significant changes were observed in cell morphology, proliferation, differentiation ability, and energy metabolism. Notably, when cultured in 3D, these changes were improved [189]. Researchers employed the engineered microtissues (EMTs) formed by Schiff base crosslinking to restore the chondrogenic potential of aged MSCs, the EMTs polysaccharides support the loading and release of the chondroinduction factor transforming growth factor β3(TGF-β3) [190]. In addition, Su and colleagues observed the proliferation and differentiation of UC-MSCs after passage 27 by using bone matrix-like scaffolds, results demonstrated that nHA/CS/PLGA scaffold effectively preserves the stemness and youth of UC-MSCs in the long-term passage [191]. Heo and colleagues improved the function of MSCs and their stem cell properties during culture with Poly-L-lysine (PLL). MSCs on PLL-coated plates exhibited a faster growth rate and upregulated expression of the stemness markers [192]. When seeded IGF-1-overexpressing senescent BM-MSCs into calcium-alginate scaffolds and incubated in a bioreactor with constant perfusion for varying periods to examine the effect of IGF-1 overexpression on the bone-forming capability of senescent BM-MSCs. IGF-1 overexpression in scaffolds increased MSCs survival, induced the expression of osteoblast markers, and enhanced the biomineralization of MSCs [165]. As the first group to describe a 3D-ECM culture system that faithfully recreated BM-MSC niches from mice and humans, Marinkovic's group was the first to describe a native 3D-ECM culture system and demonstrated that old murine BM-MSCs could be restored to bone formation capacity by culturing them on ECM synthesized by young MSCs [193]. With recent advances in tissue engineering technology, small molecules may be delivered in vivo more efficiently due to the ability to closely match the physical properties of most tissues [194]. Furthermore, tissue engineering may be able to control drug release, thereby extending the duration of cell exposure to small molecules.

### 4.5 Interplay between ‘rejuvenating’ treatments and MSC senescence

It is believed that exercise and caloric restriction (CR) can contribute to a longer life expectancy. A variety of physiological changes occur during exercise, including changes in blood flow, energy utilization, and the release of factors from muscle into the bloodstream. All of these physiological changes can affect the entire body. According to Schmidt and colleagues, short intensive exercises significantly increased the migratory activity of MSCs [195]. The study by Nucci and colleagues examines the impact of physical activity coupled with cell therapy on functional recovery after stroke. Results showed that local MSCs therapy combined with physical activity was more effective in alleviating motor dysfunction, particularly during the acute phase [196]. In a rat model of nerve crush injury, MSCs transplantation combined with cold-water swimming (CWS) improved functional outcomes more than MSCs alone or CWS alone [197]. Fisher-344 female rats who were trained nine weeks prior to myocardial infarction (MI), followed by AD-MSCs transplantation, showed improved morphological and functional LV parameters, inhibition of myocardial hypertrophy and fibrosis, and decreased proinflammatory cytokines in the myocardium as compared to sham MI rats [198]. Interestingly, CR altered the transcriptional profile of skeletal muscle in a manner similar to that of younger individuals [199]. Mammalian circadian rhythms are dominant in daily activities and physiological functions [200]. MSC proliferation and differentiation may be influenced by peripheral circadian clocks [201]. Sa Cha and colleagues found that transplantation of clock-enhanced BM-MSCs into the aging temporomandibular joint (TMJ) cavity resulted in an improvement in articular cartilage and a reduction in subchondral bone cysts [202]. As exercise and diet are disrupted with aging, it is very likely that age-associated changes have an impact on the function and regeneration of MSCs. There is still much to learn about this area, and it represents a great opportunity for research.

## 5. Potential MSCs rejuvenation strategies for age-related diseases

Research on MSCs have already progressed beyond the preclinical stage, and they have a wide range of potential applications in age-related diseases [203, 204]. A number of degenerative diseases can cause a decrease in the number and function of MSCs, and those diseases can be treated more effectively by rejuvenating aged MSCs (Table 2). For example, MSCs derived from patients with idiopathic pulmonary fibrosis (IPF) displayed increased cellular senescence and significantly reduced proliferative capacity compared with control-MSCs, thus reducing their beneficial effects in this disease. Through the downregulation of SIRT1, miR-155-5p suppressed autophagy of MSCs via the AMPK signaling pathway, resulting in the senescence of cells. A novel target of miR-199a-5p may be used to rejuvenate IPF-MSCs and enhance their therapeutic potential l[119]. Chronic kidney disease (CKD) may result in senescent cells accumulating as a result of chronic inflammation and oxidative stress. The MSCs from CKD patients showed reduced proliferation, accelerated senescence, and increased DNA damage compared to the MSCs from healthy controls. Metformin preconditioning exhibit more effectively attenuated inflammation and fibrosis as compared to untreated CKD-MSCs [156]. There are many senescent cells in synovial MSC preparations obtained from patients with osteoarthritis (OA). Pretreatment with ABT-263 significantly reduced SA-β-gal-positive cells and Bcl-2 expression and improved the function of MSCs [164]. Additionally, by evaluating a series of age markers in an OA model using uridine (a functional food derived from plants or animals), chondrocyte and MSC senescence can be alleviated in vitro [205].

In addition, there are some positive results from animal experiments. In MI, when pigment epithelium-derived factor(PEDF) expression was knocked down in BM-MSCs from old mice, MSC therapeutic efficacy was improved, and a cellular profile similar to that observed with young MSCs was induced [40]. By activating the PI3K/AKT and the MAPK/ERK pathways, ERBB4 rejuvenates senescent MSCs and enhances angiogenesis. According to a study, the ER4-senescent-MSC group showed improved blood vessel density, reduced cardiac remodeling, and reduced apoptosis four weeks after MI [206]. Infant MSC-derived EVs rejuvenated elderly MSCs from both type 1 and type 2 diabetic mice by inhibiting ROS production, accelerating cellular senescence, and promoting proliferation and in vivo functions [207]. MiR-10a overexpression activated Akt and increased the expression of angiogenic factors in senescent hBM-MSCs, thus increasing angiogenesis in ischemic mouse hearts [120]. A mouse model of myocardial infarction using hBM-MSCs downregulated by lnc-CYP7A1-1. By downregulating lnc-CYP7A1-1 in aged hBM-MSCs, MSCs are rejuvenated for heart repair therapy [131]. In aged hBM-MSCs, overexpression of NDNF decreased senescence and apoptosis. In a study, the engraftment of aged hBM-MSCs overexpressing NDNF into ischemic areas of mice's hearts improved cardiac function after myocardial infarction and enhanced the survival of the MSCs implanted [166]. These findings not only highlight the effectiveness of rejuvenation strategies in preventing MSC senescence but also open an avenue for treating age-related diseases.

## 6. Challenges and future directions

By analyzing the related research, we concluded that MSC senescence is associated with epigenomic alteration, loss of proteostasis, metabolism dysfunction, and other mechanisms, predominantly through three pathways, sirtuins/NAD^+^, AKT/mTOR, and Wnt/β-catenin which influence MSC senescence during aging. It has been determined that there are many critical points in the aging process of MSCs that may be suitable for rejuvenation. However, there are no established standards for assessing the degree of MSC senescence during aging and its impact on biological characteristics or physiological properties.

To rejuvenate MSC senescence during aging, we need a complete model and breakthrough at the molecular level. Based on current research findings, epigenetic modification, inhibition of SASP secretion, elimination of senescent cells, and improvement of MSCs culture conditions in vitro may all provide effective rejuvenation strategies and enhance the efficiency of cell transplantation therapy. In terms of current rejuvenation strategies, there are still several limitations associated with them. For example, gene modifications might result in insertional mutagenesis which may increase the risk of tumorigenesis and raise ethical concerns. Furthermore, pretreatment with biomolecules or chemicals may cause unpredictable adverse reactions in patients. In contrast, physiological preconditioning could be the breakthrough in rejuvenating aged MSCs, with relatively safe outcomes and fewer side effects. The effects of these interventions on different tissue MSCs during aging will need to be determined. With advancements in research concerning MSC senescence, the underlying mechanisms, and strategies for maintaining MSC youth and viability without causing harmful side effects will be found soon. Consequently, innervation plays a key role in modulating MSCs during aging and represents a potential therapeutic avenue. The autologous transplantation of MSCs must overcome several barriers, including culturing and transplanting MSCs at the proper time, maintaining the youthful state of the cells, and preventing tumorigenesis. It is important to determine which mechanisms are likely to provide better therapeutic opportunities-autologous MSC transplantation or manipulating endogenous MSC niches.

## 7. Conclusion remarks

It is well established that MSC transplantation therapy is a highly effective treatment for a variety of refractory diseases. Both preclinical and clinical studies have demonstrated this. However, in the case of MSCs derived from aging individuals or from long-term cultures, senescence is an inevitable biological process that limits their use in clinical settings. It is particularly important to study these findings in order to improve autologous stem cell transplantation in the elderly, who are most in need of such therapies. The understanding of the mechanisms underlying the senescence of MSCs and the aging of MSC niches may also facilitate the development of new strategies to reverse aspects of MSC senescence and reverse tissue aging. While most current research examines the therapeutic effects of MSCs on diseases, it is clear that rejuvenating the MSC senescence and renewal of senescent MSCs will be important directions for future MSCs-based clinical applications. Translating these findings into clinical therapy will contribute to the now rapidly advancing field of stem cell therapy. Ultimately, a better understanding of the causal role of MSCs in aging will be essential to the development of MSCs therapy.
